# Big 5, Little Choice? Lehren aus der Pandemie für die Regional- und Binnennachfrage nach Safaridestinationen

**DOI:** 10.1007/s00548-022-00777-3

**Published:** 2022-04-25

**Authors:** Manuel Bollmann

**Affiliations:** Global South Studies Center (GSSC), Universität zu Köln, Classen-Kappelmann-Str. 24, 50931 Köln, Deutschland

**Keywords:** Afrika, Safari, Tourismus, Reisemärkte, Kolonialismus, COVID-19

## Abstract

Auf das südliche Afrika verteilte sich vor der Pandemie circa 2 % des weltweiten internationalen Reiseaufkommens. Davon erreichten fast die Hälfte dieser jährlichen Ankünfte Südafrika. Während der Pandemie hat sich gezeigt, dass gerade die Zugpferde des internationalen Tourismusmarketing der Region, die Safarilodges und Naturerlebnisanbieter, ohne Ankünfte aus Übersee nicht zu bewirtschaften sind. Als Alternative für diesen Nachfrageeinbruch wird einer Steigerung der Regional- und Binnennachfrage kaum Potenzial zugestanden. Dieser Standpunkt soll hier bestritten werden, ohne dabei jedoch vom Alleinstellungsmerkmal des Safaritourismus abzurücken. Die Empfehlung zielt stattdessen auf eine Erweiterung des Angebotsspektrums ab. Dabei werden 3 Absichten verfolgt: Erstens, den Mangel an der Binnennachfrage für die klassischen Reiseangebote der Region zu ergründen, zweitens Chancen für eine Aufwertung des Afrikabildes darzulegen und drittens Potenziale für eine Produktdiversifizierung aufzuzeigen, die auch eine wachsende Regional- und Binnennachfrage in den Blick nimmt.

Wem die *Big 5* kein Begriff sind, lernt ihn spätestens bei der Ankunft am Tor des Safariparks kennen. Dass dieser zum Markenkern des Safaritourismus gewachsene Sammelbegriff für Leopard, Büffel, Löwe, Elefant und Nashorn der kolonialen Trophäenjagd entstammt, erfahren Reisende selten. Dass es nun ab der ersten Safari darum geht, möglichst viele dieser Tiere zu erspähen, ist aber schnell als Erfolgsziel ausgemacht. Safaritourismus ist das Alleinstellungsmerkmal Ostafrikas und des südlichen Afrikas, dem geographischen Untersuchungsgegenstand dieses Artikels. Insbesondere charismatische Säugetiere gibt es in vergleichbarem Artenaufkommen in keinem anderen Zielgebiet der Welt zu fotografieren.

Dennoch ist das südliche Afrika im globalen Vergleich nur sehr gering in internationale Reisemärkte eingebunden und auch regional ist die touristische Reisetätigkeit schwach. Mit Regionaltouristen sind hier Reisende gemeint, die sich zwischen den Staaten der Region bewegen.

Studien zu Binnentourismus für die Region sind sehr begrenzt (Rogerson 2015). Viele Länder erheben keine Daten zum Binnentourismus oder veröffentlichen sie nicht. Die wirtschaftliche Relevanz des Binnen- und Regionaltourismus steht aber laut der Welttourismusorganisation der Vereinten Nationen (UNWTO) außer Frage (UNWTO 2020). Strategien zur Förderung des Binnentourismus werden zwar immer wieder entwickelt, bisher aber selten erfolgreich umgesetzt (Florio 2020).

Internationale Tourismusankünfte auf dem Kontinent werden von globalen Statistiken in den Schatten gestellt. Gemessen an den letzten Daten der UNWTO vor dem Ausbruch der Pandemie, gab es 2019 weltweit 1,464 Mrd. internationale Touristenankünfte. Afrikas Anteil daran betrug 68,6 Mio. oder knapp 4,7 % (UNWTO 2022, S. 4). Nach den letzten präpandemischen Daten der Weltbank hat das südliche Afrika im Jahr 2019 lediglich 30 Mio. internationale Reisende empfangen. Davon entfiel fast die Hälfte auf Südafrika (World Bank 2022, o. S.).

Für die meisten Destinationen sind die Daten zu Touristenankünften in der Region jedoch höchst unzuverlässig, vor allem deshalb, weil vielerorts klandestine Arbeitsmigranten als Touristen erfasst werden. Dies betrifft nicht nur Einreise-, sondern auch Transitdaten. Überseereisende, die mit hoher Sicherheit größtenteils als Urlaubs- oder Geschäftstouristen gelten können und auch umfänglich touristische Dienstleistungen nutzen, machen deutlich kleinere Anteile unter den Ankünften aus: Diese Verteilung erstreckt sich unter den Staaten des südlichen Afrikas zwischen 12,7 % in Botsuana, einem Land mit sehr hohem Transitanteil und 28,1 % in Südafrika, dem beliebtesten Zielland für klandestine Arbeitsmigration in der Region (Fair Trade Tourism 2015, S. 7). Leider gibt es keine Studien, die eine statistische Abgrenzung zwischen regionalen Touristen und klandestinen Arbeitsmigranten nachweisen können. Trotz dieser Unsicherheiten kann man davon ausgehen, dass der Anteil der Region an der globalen Tourismusbranche bei insgesamt etwa 2 % liegt (World Bank 2022, o. S.). Die meisten Staaten in der Region sehen daher zu Recht Wachstumspotenzial für ihre Tourismusbranchen, die noch im Jahr 2017 immerhin 9 % gegenüber dem Vorjahr verzeichnen konnten (Rogerson und Baum 2020, S. 733). Dabei sollten gerade Wachstumsmärkte im Binnen- und Regionaltourismus nicht außer Acht gelassen werden (ebd., S. 735).

Das südliche Afrika war über die ersten beiden Jahre der Pandemie eine der weltweit am stärksten von Reisebeschränkungen betroffenen Regionen und wurde so fast dauerhaft von seinen wichtigsten Quellmärkten abgeschnitten. Seit Beginn der Pandemieauswirkungen im Frühjahr 2020 schätzt der World Travel & Tourism Council (WTTC 2021, S. 11), dass in ganz Afrika insgesamt fast die Hälfte des Tourismusbeitrags zum kombinierten Bruttoinlandsprodukt des Kontinents verloren und ein Drittel aller Arbeitsplätze in der Branche abgebaut wurden. Als Nebeneffekt führten diese Entwicklungen auch zu dramatischen Einnahmeverlusten für ländliche Gemeinden, die in der Nähe von Naturschutzgebieten leben und in denen der Safaritourismus vor der Pandemie die Haupteinnahmequelle war. Dasselbe gilt für Naturschutzgebiete und damit die für den Naturschutz und den Erhalt des Grundkapitals der Destinationen erforderlichen Budgets der Parkbehörden (Spenceley et al. 2021). Gerade die Binnen- und Regionalmärkte des südlichen Afrikas waren und sind weit davon entfernt diese Einbrüche aufzufangen (Van der Merwe et al. 2021).

Die Folgen der Pandemie und entsprechende Bewältigungsstrategien werden an anderer Stelle hinreichend diskutiert (Rogerson und Baum 2020; Ioannides und Gyamóthi 2020). Hier liegt der Schwerpunkt auf der Frage, wie sich der Regional- und Binnentourismus besser aufstellen könnte, um einen künftigen pandemie- oder anderweitig bedingten Nachfrageeinbruch auf den Überseemärkten besser aufzufangen.

## Big 5, Little Choice

Eine Woche Campen in Safariparks klingt spannend. Aber nach dem zweiten oder dritten Tag wiederholen sich die Abläufe. Die frühmorgendliche Begeisterung für Spähfahrten weicht irgendwann der Erinnerung an die für die Gesäßpartie unbequeme Ruckelpiste des Vorabends. Die meisten Safariparks bieten nur ein einziges Produkt an: Spähfahrten. Hinzu kommen vereinzelt Spähwanderungen und Bootsafaris, wo es die Landschaft hergibt. Das Umland der Parks ist in der Regel kaum in die Destination eingebunden. Selten gibt es im Umkreis der Parks beispielsweise Angebote für einfache und familientaugliche Wander- oder Radtouren. Mountainbikestrecken werden zwar immer öfter angeboten, allerdings sind diese häufig viel zu anspruchsvoll für den Durchschnittsreisenden. Auch Parks, die keine gefährlichen Tiere beherbergen und daher eigentlich Wanderer und Radfahrer anziehen könnten, nutzen diese Potenziale nur selten. Zusammengefasst ist das Angebot an Produktlinien im Umfeld des *Big-5-Tourismus* begrenzt (Morupisi und Mokgalo 2017). Im südlichen Afrika findet, anders als in vielen anderen Weltdestinationen, zudem wenig Vermittlung von Kulturgeschichte in Form von konkreten Inhalten und Sehenswürdigkeiten statt – obwohl diese existieren.

Die Einkommen der Hälfte aller Menschen im südlichen Afrika befinden sich noch immer unterhalb der Armutsgrenze und auch für die ausnahmslos kleinen afrikanischen Mittelschichten ist für reine Urlaubsreisen kein Geld in der Haushaltskasse. Das heißt aber nicht, dass es keine Oberschicht gibt, die Urlaubsreisen unternehmen könnte und dies nicht auch täte. Zudem beginnt sich das Bild zu wandeln, denn Reisen wird gerade durch die weite Verbreitung von Instagram und andere sozialen Medien zunehmend auch für die Pflege des sozialen Status relevant (Statista 2021; Adenkule und Kajumba 2021).

Das vorherrschende Bild im Tourismusmarketing des südlichen Afrikas und Ostafrikas spiegelt in großen Teilen auch das tatsächliche Angebotsspektrum ab: Mit Megafauna bestückte Savannenlandschaften. Abgesehen von Kapstadt und den Viktoriafällen ist das breite Angebot an Safariparks das wichtigste Zugpferd für den internationalen Reisemarkt.

Die Begeisterung für Romantik und Abenteuer der afrikanischen Wildnis und den *Big 5* wird allerdings nur von einem kleinen Teil der Binnen- und Regionalmärkte auf dem Kontinent geteilt (ebd.). Letzteres betrifft größtenteils die urbane weiße Mittel- und Oberschicht Südafrikas. Doch gerade in Südafrika wäre die Ausschöpfung eines breiteren Nachfragepotenzials auch in gehobenen Marksegmenten möglich. Im Jahr 2019 wurden dort 54 % der oberen 10 % aller Einkommen von der ehemals unterdrückten schwarzen Mehrheitsbevölkerung erwirtschaftet (Chatterjee et al. 2021, S. 33). Das naturnahe Tourismusangebot hat sich aber mit einigen Ausnahmen noch kaum auf die Bedürfnisse der in diesem Bevölkerungsteil enthaltenen Zielgruppen eingestellt.

Die Pandemie hat die Notwendigkeit aufgezeigt, den inländischen und regionalen Tourismus zu stärken, insbesondere in Naturschutzgebieten, um diese widerstandsfähiger gegen plötzliche Einbrüche der Tourismuseinnahmen aus Übersee zu machen (Rogerson und Baum 2020). Aber dafür müssen die Angebote und Produkte der Branche diversifiziert und sowohl preislich als auch inhaltlich stärker auf die Nachfrage des Inlandsmarkts ausgerichtet werden.

## Safari-Lodge-Kultur: zu Gast auf dem eigenen Kontinent?

Das Marketingdesign der Safaribranche ist auf Überseemärkte oder Afrikaner europäischer Abstammung zugeschnitten. Das zeigt sich gerade auch bei dem überwiegend hochpreisigen Safari-Lodge-Segment (ebd.). Viele Destinationen bieten den eigenen Bürgern zwar Rabatte auf die Parkgebühren an. Vergünstigte Tagesausflüge in die Parks sind in den meisten Destinationen üblich und haben seit Pandemiebeginn stark zugenommen. Doch es gibt weitere Hindernisse, die viele Einheimische von längeren Aufenthalten in den Lodges abhalten. Neokoloniale Untertöne können Einheimische abschrecken (Florio 2020). Gerade in den gehobenen Segmenten, muten diese Kulissen und Ausstattungen der Lodges an wie eine Reise in die Zeit des 19. Jahrhunderts. Doch nicht nur die Kulisse des Safarierlebnisses selbst, sondern auch Begegnungen mit der Lokalbevölkerung können befremdliche Assoziationen wecken.

Seit über 40 Jahren befasst sich die Tourismuskritik mit ungleichen Machtverhältnissen zwischen Besuchern und Besuchten (Fairunterwegs 2022). Die Kultur der Besuchten spielt beim klassischen *Big-5-Tourismus* aber nur eine Randrolle. In der Regel wird einmal im Reiseablauf zwischen Safaripark und Lodge am Wegesrand kurz Halt gemacht, um in einem eigens für diesen Zweck errichteten *Kulturdorf (Engl.: Cultural Village)* ein paar Bilder von Einheimischen vor ihren Hütten zu machen. Leider findet hier jedoch wenig Vermittlung von Kulturgeschichte in Form von konkreten Inhalten und Sehenswürdigkeiten statt, obwohl diese existieren. Kultur wird meist auf ein austauschbares Vortanzen bzw. Vorsingen der entsprechenden Volksgruppe begrenzt, in dessen Heimat man sich gerade befindet. Was für manch einen Überseetouristen wohl als halbwegs authentische Darstellung der lokalen Kultur durchgehen mag, kann für Menschen, die selbst aus diesen oder verwandten Kulturgemeinschaften kommen, zumeist vor allem eines sein: peinlich.

In dieser Form sind *Kulturdörfer* sowohl für die Protagonisten als auch für Binnentouristen nicht gerade zweckdienlich, um eine Neugier danach zu wecken, mehr über die eigene Kultur oder die Kultur anderer Landsleute zu erfahren. Manche dieser *Kulturdörfer* erinnern weniger an zeitgenössische afrikanische Kulturen, sondern scheinen eher in der rassistischen Kolonialtradition der Völkerschauen verwurzelt zu sein. Als der Augsburger Zoo 2010 auf die Idee kam, ein solches für Touristen konstruiertes *Kulturdorfkonzept *mal auf dem eigenen Gelände in der Stadt auszuprobieren, lösten die Veranstalter einen Sturm der Entrüstung aus (SZ 2010). Dabei ist das eigentliche Problem sowohl im Falle Augsburgs als auch bei dem „Original“ der *Kulturdörfer* in Afrika weniger das räumliche Nebeneinanderstellen von Menschen und in Gefangenschaft lebender Tiere, sondern die inhaltliche Assoziation und das Gleichsetzen eines kolonialen auf „Wildnis“ reduzierten Afrikabildes mit heute lebenden Menschen und ihren Kulturen (Chikha und Arnaut 2013).

Abgerundet wird das koloniale Bild von Besuchern und Besuchten von den Besitzern der meisten Safariunternehmen: Ein großer Teil der Branche befindet sich weiterhin in der Hand von Männern, die den *Old Boy Networks* entstammen, deren Ursprung in die Zeit der Apartheid zurückreicht (Jänis 2014). Nicht zu Unrecht haben einige afrikanische Interessenvertreter im Zuge einbrechender Überseeankünfte seit Beginn der Pandemie eine stärkere Ausrichtung touristischer Angebote auf die Binnennachfrage angeregt und in Zuge dessen teilweise auch eine *Dekolonialisierung* des Safaritourismus gefordert (Florio 2020).

## Die Pandemie als Chance, Geschichte(n) neu zu schreiben

**Abb. 1:**
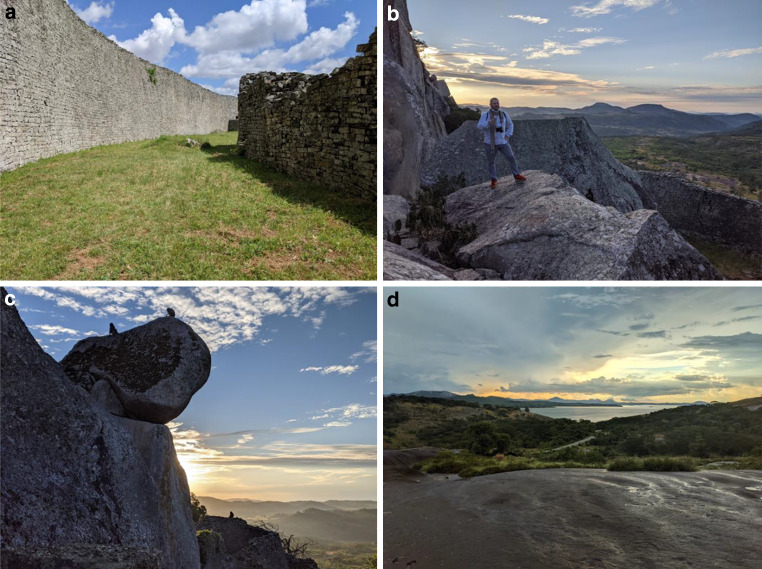
Kerndestination Groß-Simbabwe (**a**) und ihr umliegendes Landschaftsbild (**b**–**d**). (Bildrechte: Manuel Bollmann)

Neben der Diversifizierung von Naturerlebnisprodukten wäre ein weiterer Schritt in die richtige Richtung die Stärkung des Kulturerbetourismus, der die schablonenhafte Aufstellung von Kulturdörfern überwinden kann, sich explizit auch an Regional- und Binnentouristen wendet und statt Peinlichkeiten nicht nur neues Interesse, sondern auch Neugier und Stolz auf die eigene Geschichte, Bräuche und Traditionen fördert (Cole 2007, Mbaiwa 2011). Namibia hat gerade eine Strategie für nachhaltigen Kulturtourismus (MET 2021) auf den Weg gebracht. Botsuana fördert den Agrotourismus, der als Konzept bäuerliche Gemeinden und deren tradierte Lebensweisen einbezieht (Tourism Update 2020). Doch bevor Angebote für Produktlinien im Bereich Kultur und Geschichte etabliert werden, müssen sie ausgestaltet werden. Dies betrifft sowohl die Inwertsetzung kulturhistorischer Destinationen durch entsprechende Infrastruktur und die Entwicklung moderner Konzepte für Führungen als auch breitgefächerte Zusatzangebote im Umfeld der Hauptattraktion. Ein Paradebeispiel ist Groß-Simbabwe im Süden Simbabwes, dessen Ruinen zwar mit teilweise gut ausgebildeten Museumsführern besucht werden können, deren welthistorische Bedeutung als hochmittelalterliches Handelszentrum für Gold und Elfenbein aber kaum vermittelt wird. Auch wird bisher das für die touristische Nutzung hochattraktive Umland von Groß-Simbabwe kaum in Wert gesetzt oder mit vermarktet (Abb. 1).

Ein Bewusstsein für die Rolle der sozialen Medien für die Ausgestaltung von Marketingnarrativen im Tourismussektor ist zwar längst in der Region vorhanden. Abgesehen von Südafrika, das auch in diesem Bereich eine Vorreiterrolle auf dem gesamten Kontinent spielt, gibt es aber bei Destinationen, die sich auch jenseits der *Big 5* positionieren, noch viel zu tun. Das betrifft sowohl die Narrative (*Storytelling*) als auch das Einbettungspotenzial von Narrativen in Destinationskulissen (*Instragrammability*). Letztere sind wichtige Faktoren für Reiseentscheidungen, gerade für afrikanische Quellmärkte, die fast ausschließlich aus jüngeren Generationen bestehen (Gumpo et al. 2020).

Südafrikas Marketingstrategie und -kampagne für den Inlandstourismus „Sho’t left“ (Rogerson und Lisa 2005) ermutigt Reisende im wörtlichen und übertragenen Sinne, einmal von ihrem Wohnort aus „kurz links“ abzubiegen und Reiseangebote in ihrem eigenen Land und den umliegenden Gemeinden zu erkunden. Während die Entwicklungen zur Förderung des Inlandstourismus in Namibia und Botsuana noch relativ jung sind, bietet „Sho’t left“, das bereits 2004 ins Leben gerufen wurde, viele Lehren dafür, wie man neue Märkte unter der eigenen Bevölkerung erschließen kann.

Erfolgreich sind vor allem Produkte, die kompatibel mit Gruppeninteressen, zeitlich begrenzt und nicht zu kostspielig sind. Zielgruppen können Familienverbünde sein, aber auch Freundesgruppen oder Liebespaare. Stattdessen werben viele Destinationen, beispielsweise die Viktoriafälle oder die Drakensberge, mit aufwendigen Abenteuerprodukten, die für regionale Märkte nicht nur deutlich zu viel Nervenkitzel ankündigen, sondern auch leere Brieftaschen.

## Fazit

Letztlich haben wir es bei der vorgestellten Thematik im Wesentlichen mit 3 Trends zu tun: ein beispielloser Abbruch interkontinentaler Ankünfte in der Region über einen Zeitraum von anderthalb Jahren; ein etablierter Big-5-Tourismus, der sein Erscheinungsbild auf eine kleine aber vergleichsweise zahlungskräftige Zielgruppe ausrichtet und sich dabei kolonialer Designs bis hin zu einem *Reenactment* kolonialer Rollenbilder bedient; und eine aufstrebende afrikanische Mittel- und Oberschicht, die neue Reisebedürfnisse hat, die mehr mit einer digitalisierten Moderne als mit der Ästhetik des ausgehenden 19. Jahrhundert zu tun hat.

Natürlich ist es schwierig bis unmöglich nach 2 langen verlustreichen Geschäftsjahren in neue Angebote und Ausstattungen zu investieren. Es ist leicht nachzuvollziehen, dass eine Angleichung von Übernachtungspreisen an regionale Einkommen auch zu Qualitätseinbußen im Angebot führen kann. Doch diese Einschätzungen sind buchstäblich zu kurz gedacht. Der Safarisektor ist gut beraten, an seinen Kernangeboten und Märkten festzuhalten. Zugleich sollte der Sektor sich konzeptionell öffnen, indem er zumindest beginnt, mit neuen Produktlinien zu experimentieren und eine inklusivere Zielgruppenstrategie zu fahren. Destinationen sollten kulturelle und historische Angebote nicht nur stärker ausbauen, sondern diese inhaltlich auch mit marktorientiertem *Storytelling* ausgestalten. Langfristig bedarf es einer Dekolonialisierung der Eigentumsverhältnisse touristischer Betriebe. Zur Aufwertung der Erlebnisprodukte ist aber dringend auch eine deutliche Aufwertung kultureller Reiseinhalte durch die Inhaber des kulturellen Eigentums selbst gefragt.

Sowohl das Afrikabild in Übersee als auch die Reisebereitschaft in den Binnen- und Regionalmärkten Afrikas befinden sich in einem Wandel, der nach der weltweiten Eindämmung der Pandemie und einer hoffentlich damit einsetzenden wirtschaftlichen Erholung in der Region, an Dynamik hinzuzugewinnen verspricht. Um auf diesen Zug aufzuspringen, bedarf es neben den *Big 5 *auch an *Bigger Ideas*.
